# Effectiveness of a field trap barrier system for controlling *Aedes albopictus*: a “removal trapping” strategy

**DOI:** 10.1186/s13071-018-2691-1

**Published:** 2018-02-20

**Authors:** Mohammad Akhoundi, Frédéric Jourdain, Fabrice Chandre, Pascal Delaunay, David Roiz

**Affiliations:** 1 Service Parasitologie-Mycologie, Hôpital de l’Archet, CHU de Nice, Nice, France; 2 MIVEGEC, UMR IRD224-CNRS5290-Université de Montpellier, Montpellier, France; 3 Centre National d’Expertise sur les Vecteurs, Montpellier, France

**Keywords:** Vector control, Removal method, Trap barrier system, CO_2_ baited trap, *Aedes albopictus*

## Abstract

**Background:**

*Aedes aegypti* and *Aedes albopictus* are the main vectors for the transmission of several viral pathogens, in particular, dengue, Zika and chikungunya. In the absence of vaccines and treatment, control of *Aedes* mosquitoes is the only means of keeping these diseases in check. *Aedes* control is difficult, and it is, therefore, necessary to evaluate the efficacy of novel control methods, particularly those targeting adult and exophilic *Ae. albopictus* populations.

**Methods:**

We carried out the first evaluation of the effectiveness of a field trap barrier system, i.e. a “removal trapping” outdoor control strategy for *Ae. albopictus* in southern France.

**Results:**

The removal trapping control strategy is an effective system, able to reduce to almost zero the biting rate of the tiger mosquito in and around houses with traps installed. This strategy has the advantage of being a non-chemical method, which is environmentally friendly and does not affect non-target fauna. Nevertheless, it has several constraints including the cost of the CO_2_ required for the system to function. However, the system could be optimized by reducing the costs and combining it with other control strategies within the framework of integrated vector management.

**Conclusions:**

We provide the first evidence of the effectiveness of this trap barrier system, which is based on the combined effect of (i) removing adult mosquitoes living in the area, and (ii) hampering the migration of mosquitoes from outside into the treated area. Further investigation is needed to understand its efficacy for other species, other locations and at-risk communities, and to evaluate its application for reducing the prevalence of dengue, Zika and chikungunya diseases.

**Electronic supplementary material:**

The online version of this article (10.1186/s13071-018-2691-1) contains supplementary material, which is available to authorized users.

## Background

Vector-borne diseases are among the leading causes of mortality and morbidity in humans, with more than one billion people infected and more than one million deaths per year [1]. Increasing travel and trade over recent decades and uncontrolled urbanization have made this a global threat and have escalated the disease burden and risk. At present, the emergence of the Zika virus along with increasing incidences of dengue and chikungunya outbreaks have made *Aedes*-borne disease control and surveillance a public health priority [2].

The etiological agents of these diseases, mainly transmitted by *Aedes aegypti* and *Aedes albopictus*, have emerged not only in tropical regions but also in temperate areas, due mainly to the spread of *Ae. albopictus* [3]. This species has been established in France since 2004, primarily on the Mediterranean coastline, including Nice. The populations of *Ae. albopictus* from this area were shown to be competent for transmission of chikungunya and dengue viruses under laboratory conditions [4] and, to a lesser extent, for Zika and yellow fever viruses [5, 6]. *Aedes albopictus* may be present at high densities and is responsible for several autochthonous cases of dengue and chikungunya in southern France [7–12]. It has also been reported as the vector of several outbreaks of dengue and chikungunya viruses in other European countries [13–15]. To reduce the risk of *Aedes-*borne viruses and mosquito nuisances in Europe, it is of prime importance to control *Ae. albopictus* populations.

Controlling the *Ae. albopictus* population is a difficult and complex task, as these mosquitoes can breed in ephemeral and cryptic containers, which are ubiquitous in domestic environments [16, 17]. Recent reviews of the evidence for the effectiveness of *Aedes* control strategies (including against *Ae. albopictus*) show them to be usually low [18]. Chemical control using insecticides has been the main strategy for controlling adult *Aedes* populations over the last 60 years, but it has numerous drawbacks including insecticide resistance, environmental contamination, bioaccumulation of toxins, impact on non-target fauna and limited acceptability. There is, therefore, an urgent need to find new effective strategies that target adult populations [19] and, especially for *Ae. albopictus*, with a predominant exophilic fraction of the populations [3].

In light of all this, it is of prime importance to investigate new mosquito control methods for the future. These methods should be complementary to current strategies and preferably (i) target adult populations, in particular, the host-seeking females (even if larval control is in priority); (ii) use non-chemical insecticides, given the emergence of a new generation (or population) of insecticide-resistant mosquitoes; and (iii) be species-specific to avoid negative impacts on non-target organisms and, more broadly, on the environment.

Removal trapping involves the use of target-specific attractants to lure large numbers of a specific insect species and then kill them to reduce/eliminate a population in a pre-defined area [20]. This strategy has been used successfully against *Hippelates* gnats in the USA [21], tsetse flies in West Africa [22–24], *Stomoxys calcitrans* in Australia [25] and tabanids in the USA [26]. It has been a solution for eliminating the insect vectors of sleeping sickness in many foci in West Africa [24].

Few studies have been conducted on the use of trap barrier systems to control mosquitoes, although mention should be made of the various experiments that have been carried out since 1996 to control salt-marsh mosquitoes (*Ochlerotatus taeniorhynchus*) in Florida [27] and in the Gulf of Mexico [28].

Despite these investigations, a few studies have reported these barrier traps as being ineffective in controlling mosquitoes [29]. A review of these studies was carried out in the past [20, 28] and, to our knowledge, no new experiments have been recently carried out with mosquitoes. A similar control strategy with the wide use of BG-Sentinel traps (Biogents AG, Regensburg, Germany) - but not as a trap barrier system - was used for mass trapping of *Aedes aegypti* in Brazil [30]. Recently, intervention with BG-Sentinel traps in conjunction with BG-Lure has resulted in a reduction in *Ae. albopictus* biting pressure in Italy [31].

This study aimed to evaluate the effectiveness of a new trap barrier system in reducing the biting rate of *Ae. albopictus* in individual houses during seasonal peak activity.

## Methods

**Fig. 1:**
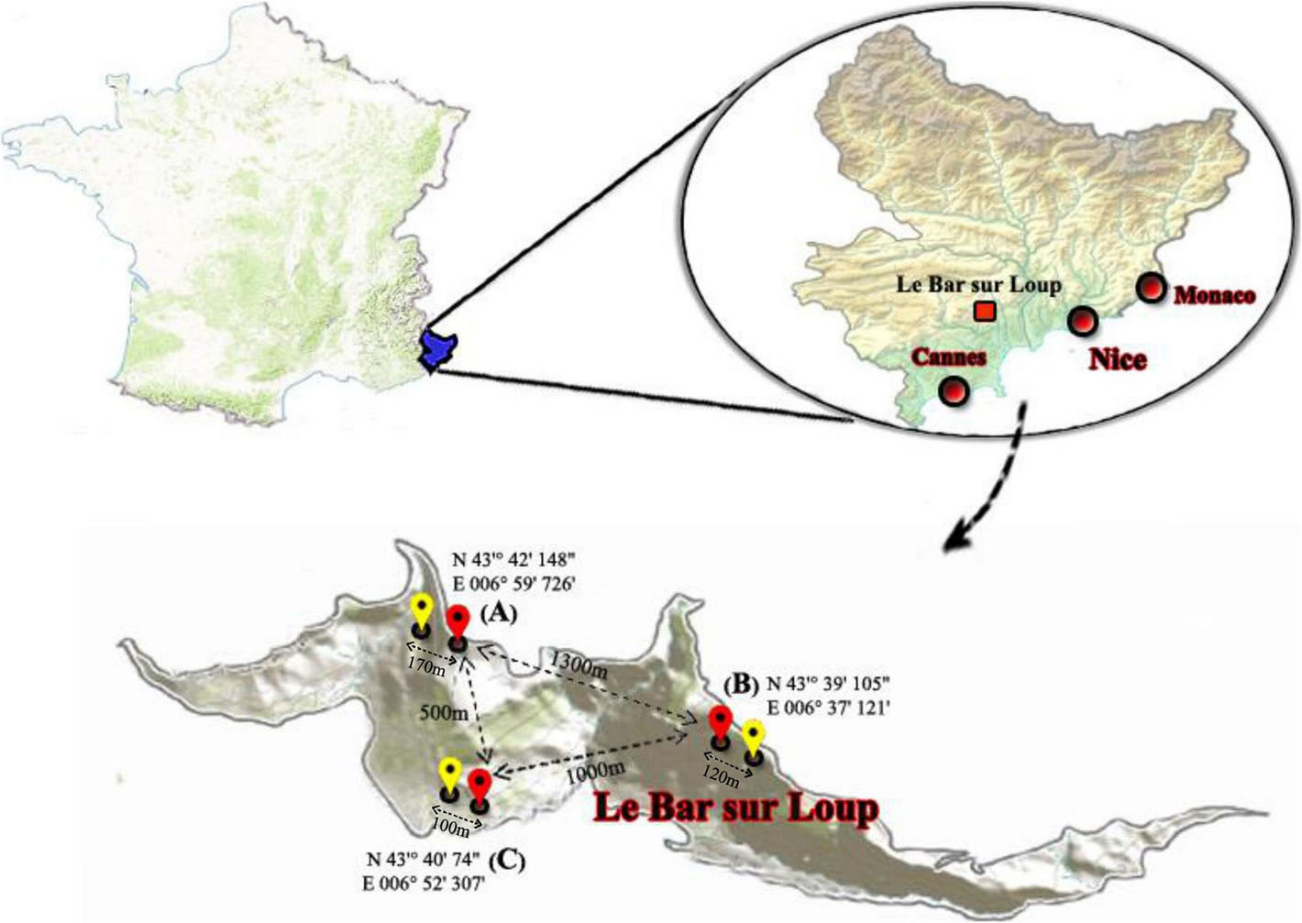
Geographical positions of treated (*red*) and control (*yellow*) houses in the present study

The study was conducted from July to September 2016 in residential areas of the village of Le Bar-sur-Loup in the Provence-Alpes-Côte d’Azur, France, which includes 800 houses and has 1447 km^2^ surface and an average altitude of approximately 300 m above sea level (Fig. 1).

**Fig. 2:**
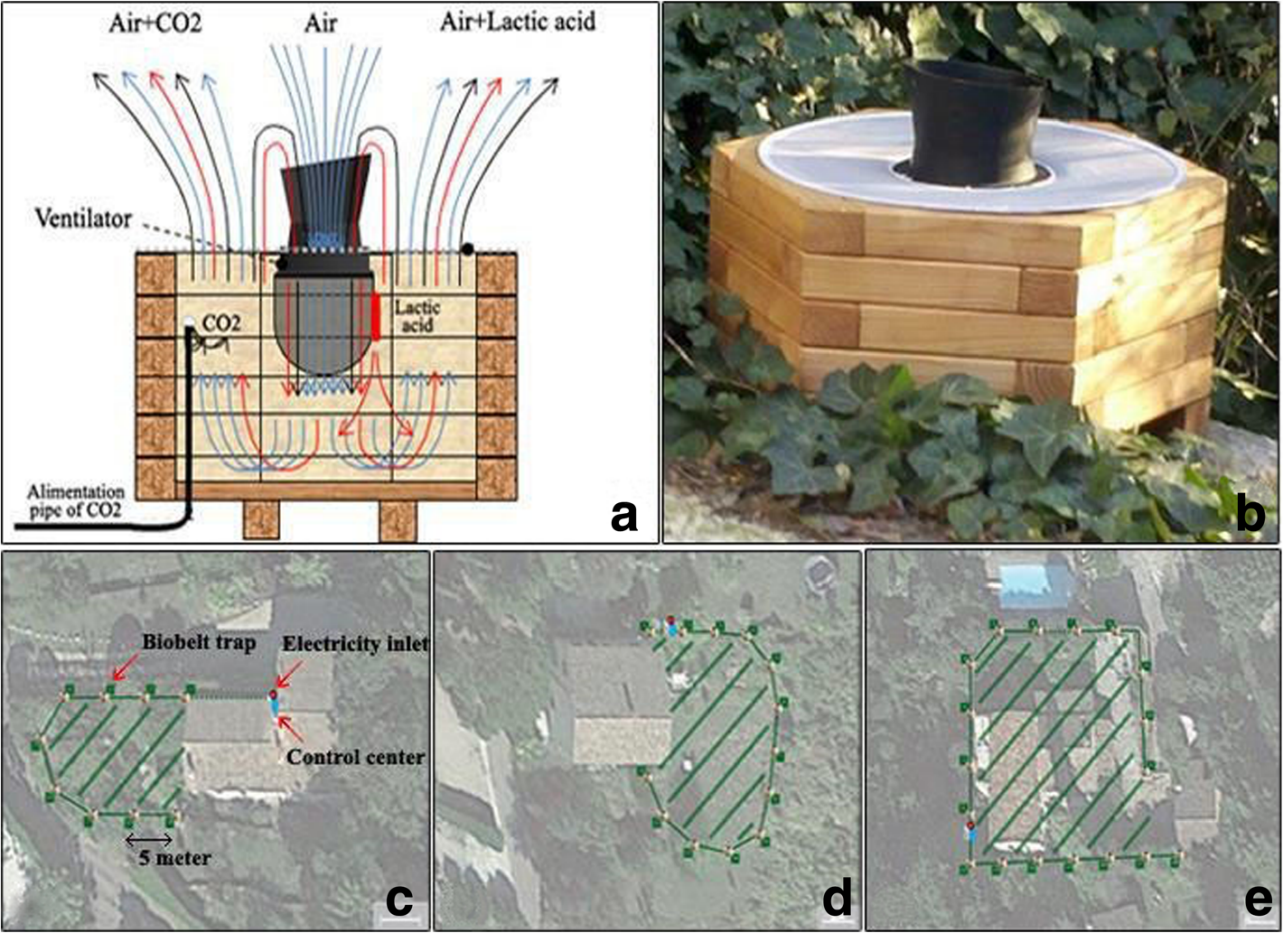
Biobelt traps installation in the treated houses. **a** Scheme of the trap function, based on the Biogents mosquito traps. **b** Photo of an individual trap. **c**-**e** Spatial schematic depiction of the array of the “belt” of traps around three treated houses, prospected in the area Modules-traps are shown in green, control center in blue and electricity inlet in red. The area protected by the barrier is hatched

To evaluate the efficacy of the trap barrier system, we selected three pairs of houses (each pair comprising one treated and one control house) with similar ecological and geographical environments and altitudes (Fig. 1). To choose the treated/control houses, *Ae. albopictus* populations were monitored with ovitraps (3 per house) during the 3 weeks prior to this experiment. Based on this monitoring, three classes were established according to egg density including low, medium and high with two sites in each class. Then, for each class a treated and a control site was randomly chosen. The residents were fully informed of the test procedures and accepted by official affirmation to participate in the study for 3 months during *Ae. albopictus* peak activity in this area [32]. Then, HLR (human landing rate) was performed during 2 weeks in the absence of the trap barrier (1st and 2nd weeks). The trap barrier systems were installed at the treated houses at the end of week 2. The system, BioBelt Anti-Moustiques, is a commercial trap system developed and patented by the French company HBM Distribution SAS. It consists of a network of traps positioned at an average of 5 m distance around the area to be protected and connected to a control center with a programming unit equipped with 34 kg bottles of CO_2_ and electricity supply for the trap fans (Fig. 2a, b). Individual traps use the Biogents mosquito trapping technology [33]. The number and arrangement of traps depended on the size and configuration of the treated houses and the vegetation surrounding them. This resulted in 9, 13 and 18 traps installed to protect the three experimental houses (Fig. 2c-e, respectively). Each array of traps was configured as a belt surrounding the area of the garden and the inhabited house. Mosquitoes were attracted to the traps by CO_2_ and BG-lure attractants, which have a synergistic effect that maximizes mosquito catches [32, 34]. Each trap releases CO^2^ uninterruptedly in a discontinuous dispersion cycle at a rate of 20 g/h for 10 s followed by a 10 s pause, giving an average release rate of 10 g/h. For each house, the installation of the trap barrier system was made of several elements: the traps, the electrical system and CO_2_ connections, the programming unit, a 12 V transformer, and CO_2_ bottles. Twelve, 16 and 23 h of a technicians work time were devoted to the installation of the system. Around 630 h of emission of CO_2_ per house were used during this study, yielding a total of 6.3 kg of CO_2_ per trap, and as a result 56.7, 81.9 and 113.4 kg of CO_2_ for each house, respectively.

Human landing rate (HLR) of *Ae. albopictus* in each of the treated/control houses was measured trough collecting mosquitoes by a single volunteer, the first author of this study (MA). Naked legs and hands were exposed to the bites of *Ae. albopictus* for 30 min in each house-yard corresponding to the area used by owners for outdoors activities, inside the trap barrier in the case of treated houses. Human landing catch (HLC) sessions (4 sites per day) took place daily before sunset (17:00 to 21:00 h) [35]. Mosquitoes were captured by electric mosquito killer racket, which is appropriate for active mosquitoes, such as *Ae. albopictus.* This method has the advantage that it avoids miscounting HLC due to multiple interrupted blood feeding. Moreover, it is easier to capture *Ae. albopictus* than with a mouth aspirator. Each working day, 4 sites were sampled, according to a randomization sampling. Each site was sampled 42 times during 13 weeks, with a total of 252 sampling sessions. Every week, the numbers of adult mosquitoes caught by the traps were counted and the species identified under a stereomicroscope. Daily temperature and humidity were recorded at each site throughout the study period by data loggers (1/house).

After devising a protocol for data exploration [36], statistical analysis of treatment efficacy was carried out using a generalized linear mixed model (GLMM) with negative binomial distribution as the data were over-dispersed using the automatic differentiation model builder (*glmmADMB*) package [37]. The response variable was the human landing catch, the explanatory variable control/treatment and the random variable ‘house’. Statistical analysis was performed with the R software version 3.2.2.

## Results

**Fig. 3:**
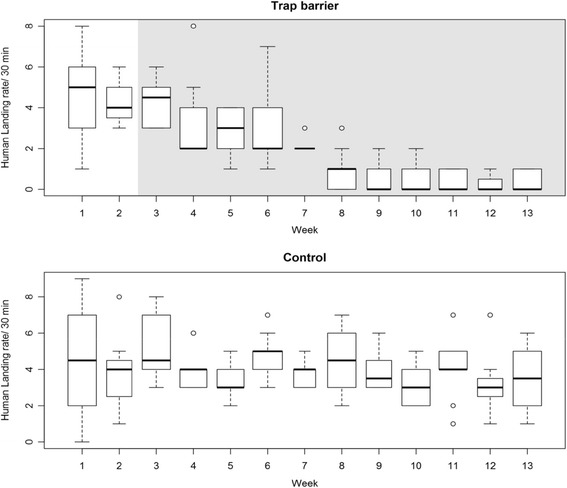
Human landing rates at treated (Biobelt trap barrier) and control houses before and after installation (grey filled) of the trap barrier

**Fig. 4:**
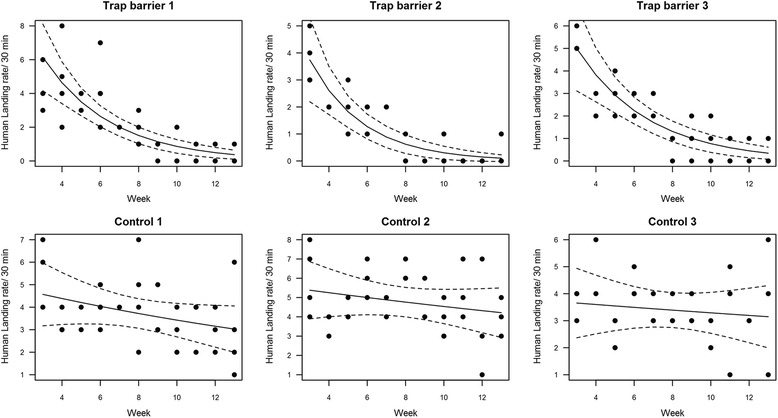
Human landing rates at each of the treated (Biobelt trap barrier) and control houses after the beginning of the treatment. The curves represent the results of a generalized linear model with negative binomial distribution

The trap barrier system was highly efficient in reducing the *Ae. albopictus* biting rate to almost zero at the treated houses 6 weeks after the beginning of the intervention (Fig. 3). The differences between the treated and control houses in human biting rates were highly statistically significant in the negative binomial GLMM analysis (*Z* = -7.65, *P* < 0.0001). A progressive diminution to 50% was observed in the first week, a further reduction by half in the fifth week, and a progressive reduction to zero bites in the 6th week and up to the end of the experiment. These gradual reductions were consistent in all the houses with trap barriers (Fig. 4). In contrast, the biting rate in all the control (non-treated) houses was constant throughout the study, with some weekly variations due to other environmental factors (Figs. 3 and 4).

Most of the insects caught by the traps (94%) were *Ae. albopictus* mosquitoes, followed by moths (4%) and *Culex pipiens* (2%). In this experiment, the system does not, therefore, affect non-target fauna, in particular, pollinators. The results of the trap catches are presented in the Additional file 1: Fig. S1. The variations in daily temperature and relative humidity recorded by the data loggers are shown weekly in Additional file 2: Fig. S2. The database of the results is included as Additional file 3: Table S1.

## Discussion

We have shown the barrier trap system to be effective in reducing to almost zero the biting rate of *Ae. albopictus* with semi-individual protection in an enclosed environment. At a time where there is evidence of the low effectiveness of some *Aedes* control strategies [11, 18, 38–41], this method could represent a promising controlling tool of *Ae. albopictus* for specific areas. The attractant-baited barrier, sometimes referred to as removal trapping, was first used with insecticides applied by aerial spraying or hand-held equipment to control tsetse flies and salt-marsh mosquitoes [28] as an alternative method of controlling adult populations. In this study, we have demonstrated, for the first time, its effectiveness in reducing human contact with *Ae. albopictus*.

The method is effective in eliminating *Ae. albopictus* mosquitoes in the treated area and represents clear benefits as a non-chemical, environmentally friendly strategy with species-specific application. Nevertheless, it has some constraints. First, the method needs some time (weeks) before it becomes completely effective and succeeds in eliminating the mosquito population within the barrier system. A possible solution to this problem would be to install the trap barrier at the beginning of the mosquitoes’ active season, thereby preventing development of the mosquito population within the barrier system, and combining with other control methods, i.e. the ovitraps. Second, CO_2_ is expensive, and therefore, the ways of reducing released amounts need to be explored (around 90 kg of CO_2_/house were used here). In addition to the cost of the CO_2_, the cost in electricity, materials, and personnel must be added. Therefore, at present, it would be difficult to generalize this method as an on-going control tool in a routine manner. To optimize its efficiency, the key challenge is to reduce CO_2_ production costs and/or consumption while maintaining efficacy. It should also be implemented in an ongoing integrated vector management strategy synergically with other control methods targeting the immature population (source reduction, environmental management, larviciding), and any other sustainable adult mosquito control method. Promising results may also be obtained by combining this method with other tools targeting the gravid population, such as autocidal gravid traps (AGO) or gravid *Aedes* traps (GAT), which have already shown encouraging results [42, 43]. The trap barrier system could also be used to protect sensitive areas, such as schools, retirement homes or hospitals.

This work raises several research questions regarding improvements to these kinds of vector control methods. There is, for example, a need to investigate the re-colonization dynamic of the study area by *Ae. albopictus* after removing the traps. Moreover, the capture effectiveness of each module of the barrier as a function of its micro-environmental conditions needs to be better understood. Proof of concept should also be demonstrated for other species, in particular, more endophilic species such as *Ae. aegypti*. Also, it is of importance to evaluate the effects of the barrier system on the disease prevalence (dengue, Zika, chikungunya) as well as biting rate. To make this barrier trap viable for use on a larger scale, a reduction in operating costs could be made by decreasing CO_2_ use, either by reducing the flow into each trap or by increasing the distance between each trap.

## Conclusions

We have shown that among the various techniques for controlling exophilic adult *Aedes* populations, barrier traps are an effective, odourless and eco-friendly method for reducing *Ae. albopictus* biting rates and possibly other mosquito species in a given area. Further investigations are needed to optimize application of this promising method and to evaluate its performance in other locations, including at-risk communities, and with other hematophagous vectors. Further studies aimed at increasing the impact of *Aedes* control interventions are needed, and guidelines and strategies need to be developed for reducing the burden of arboviruses diseases. We are optimistic that once it is improved and combined with other tools, this strategy will contribute to the panel of new methods that will open a new era of successful *Aedes* control, and consequently the control of arboviruses and other vector-borne diseases.

## Additional files

Additional file 1: **Figure S1.** Variations in daily captures of *Ae. albopictus* among traps. (TIFF 102 kb)

Additional file 2: **Figure S2.** Variations in daily temperature and relative humidity recorded weekly by data loggers. (TIFF 111 kb)

Additional file 3: **Table S1.** Database of the results analyzed in the present study. (XLSX 22 kb)

## Abbreviations

AGO: autocidal gravid traps
GAT: gravid *Aedes* traps
GLMM: generalized linear mixed model
glmmADMB: automatic differentiation model builder
HLC: human landing catch
HLR: human landing rate

## References

[1] World Health Organization (2014). A global brief on vector-borne diseases.

[2] Mayer SV, Tesh RB, Vasilakis N. The emergence of arthropod-borne viral diseases: A global prospective on dengue, chikungunya and Zika fevers. Acta Trop. 2017;166:155–63. 10.1016/j.actatropica.2016.11.020 PMC5203945 27876643

[3] Paupy C, Delatte H, Bagny L, Corbel V, Fontenille D (2009). *Aedes albopictu*s, an arbovirus vector: from the darkness to the light. Microbes Infect.

[4] Vega-Rua A, Zouache K, Caro V, Diancourt L, Delaunay P, Grandadam M, Failloux AB (2013). High efficiency of temperate *Aedes albopictus* to transmit chikungunya and dengue viruses in the Southeast of France. PLoS One.

[5] Amraoui F, Vazeille M, Failloux AB (2016). French *Aedes albopictus* are able to transmit yellow fever virus. Euro Surveill..

[6] Jupille H, Seixas G, Mousson L, Sousa CA, Failloux AB (2016). Zika virus, a new threat for Europe?. PLoS Negl Trop Dis.

[7] Delaunay P, Jeannin C, Schaffner F, Marty P (2009). News on the presence of the tiger mosquito *Aedes albopictus* in metropolitan France. Arch Pediatr.

[8] Delaunay P, Mathieu B, Marty P, Fauran P, Schaffner F (2007). Chronology of the development of *Aedes albopictus* in the Alpes-Maritimes Department of France, from 2002 to 2005. Med Trop.

[9] La Ruche G, Souarès Y, Armengaud A, Peloux-Petiot F, Delaunay P, Desprès P (2010). First two autochthonous dengue virus infections in metropolitan France, September 2010. Euro Surveill..

[10] Boubidi SC, Roiz D, Rossignol M, Chandre F, Benoit R, Raselli M (2016). Efficacy of ULV and thermal aerosols of deltamethrin for control of *Aedes albopictus* in Nice, France. Parasit Vectors.

[11] Succo T, Leparc-Goffart I, Ferré J, Roiz D, Broche B, Maquart M (2016). Autochthonous dengue outbreak in Nîmes, South of France, July to September 2015. Euro Surveill..

[12] Delisle E, Rousseau C, Broche B, Leparc-Goffart I, L'Ambert G, Cochet A (2015). Chikungunya outbreak in Montpellier, France. Euro Surveill.

[13] Jourdain F, Roiz D, Perrin Y, Grucker K, Simard F, Paupy C (2015). Entomological factors of arboviruses emergences. Transfus Clin Biol.

[14] Angelini R, Finarelli AC, Angelini P, Po C, Petropulacos K, Macini P, et al. An outbreak of chikungunya fever in the Province of Ravenna, Italy. Euro Surveill. 2007;12(9):E070906. 10.2807/esw.12.36.03260-en 17900424

[15] Gjenero-Margan I, Aleraj B, Krajcar D, Lesnikar V, Klobučar A, Pem-Novosel I (2011). Autochthonous dengue fever in Croatia, August-September 2010. Euro Surveill.

[16] Faraji A, Unlu I (2016). The eye of the tiger, the thrill of the fight: effective larval and adult control measures against the Asian tiger mosquito, *Aedes albopictus* (Diptera: Culicidae), in North America. J Med Entomol.

[17] Baldacchino F, Caputo B, Chandre F, Drago A, della Torre A, Montarsi F, Rizzoli A (2015). Control methods against invasive *Aedes* mosquitoes in Europe: a review. Pest Manag Sci.

[18] Bouzid M, Brainard J, Hooper L, Hunter PR. Public health interventions for *Aedes* control in the time of Zika virus - a meta-review on effectiveness of vector control strategies. PLoS Negl Trop Dis. 2016;10(12):e0005176. 10.1371/journal.pntd.0005176 PMC5142773 27926934

[19] Morrison AC, Zielinski-Gutierrez E, Scott TW, Rosenberg R (2008). Defining challenges and proposing solutions for control of the virus vector *Aedes aegypti*. PLoS Med.

[20] Day JF, Sjogren RD (1994). Vector control by removal trapping. Am J Trop Med Hyg.

[21] Mulla MS, Axelrod H, Wargo MJ (1990). Chemical attractant formulations against the eye gnat *Hippelates collusor* (Diptera: Chloropidae). Bull Soc Vector Ecol.

[22] Morris KRS, Morris MG (1949). The use of traps against tsetse in West Africa. Bull Entomol Res.

[23] Vale GA, Hall DR (1985). The use of l-octen-3-o1, acetone and carbon dioxide to improve baits for tsetse flies, *Glossina* spp. (Diptera: Glossinidae). Bull Entomol Res.

[24] Laveissiere C (1988). Les glossines. Guide de formation et d’information. Série lutte antivectorielle.

[25] Rugg D (1982). Effectiveness of Williams traps in reducing the numbers of stable flies (Diptera: Muscidae). J Econ Entomol.

[26] Wall WJ, Doane OW (1980). Large scale use of box traps to study and control saltmarsh greenhead flies (Diptera: Tabanidae) on Cape Cod, Massachusetts. Environment Entomol.

[27] Kline DL, Lemire GF (1998). Evaluation of attractant-baited traps/targets for mosquito management on Key Island, Florida, USA. J Vect Ecol.

[28] Kline DL (2007). Semiochemicals, traps/targets and mass trapping technology for mosquito management. J Am Mosq Control Assoc.

[29] Smith JP, Cope EH, Walsh JD, Hendrickson CD (2010). Ineffectiveness of mass trapping for mosquito control in St. Andrews State Park, Panama City Beach, Florida. J Am Mosq Control Assoc.

[30] Degener CM, Eiras AE, Azara TM, Roque RA, Rösner S, Codeço CT (2014). Evaluation of the effectiveness of mass trapping with BG-sentinel traps for dengue vector control: a cluster randomized controlled trial in Manaus, Brazil. J Med Entomol.

[31] Englbrecht C, Gordon S, Venturelli C, Rose A, Geier M (2015). Evaluation of BG-Sentinel trap as a management tool to reduce *Aedes albopictus* nuisance in an urban environment in Italy. J Am Mosq Control Assoc.

[32] Roiz D, Boussès P, Simard F, Paupy C, Fontenille D. Autochthonous chikungunya transmission and extreme climate events in southern France. PLoS Negl Trop Dis. 2015;9(6):e0003854. 10.1371/journal.pntd.0003854 PMC4469319 26079620

[33] Maciel-de-Freitas R, Eiras AE, Lourenço-de-Oliveira R (2006). Field evaluation of effectiveness of the BG-Sentinel, a new trap for capturing adult *Aedes aegypti* (Diptera: Culicidae). Mem Inst Oswaldo Cruz.

[34] Pombi M, Jacobs F, Verhulst NO, Caputo B, della Torre A, Takken W (2014). Field evaluation of a novel synthetic odour blend and of the synergistic role of carbon dioxide for sampling host-seeking *Aedes albopictus* adults in Rome, Italy. Parasit Vectors.

[35] EID. Surveillance du moustique *Aedes albopictus* en France métropolitaine. Bilan. 2007; EID Mediterranée.

[36] Zuur AF, Ieno EN, Elphick CS (2010). A protocol for data exploration to avoid common statistical problems. Methods Ecol Evol.

[37] Bolker BM, Brooks ME, Clark CJ, Geange SW, Poulsen JR, Stevens MHH, White JSS (2009). Generalized linear mixed models: a practical guide for ecology and evolution. Trend Ecol Evol.

[38] Abramides GC, Roiz D, Guitart R, Quintana S, Guerrero I, Giménez N (2010). Effectiveness of a multiple intervention strategy for the control of the tiger mosquito (*Aedes albopictus*) in Spain. Trans R Soci Trop Med Hyg.

[39] Chandel K, Suman DS, Wang Y, Unlu I, Williges E, Williams GM, Gaugler R (2016). Targeting a hidden enemy: pyriproxyfen autodissemination strategy for the control of the container mosquito *Aedes albopictus* in cryptic habitats. PLoS Negl Trop Dis.

[40] Esu E, Lenhart A, Smith L, Horstick O (2010). Effectiveness of peridomestic space spraying with insecticide on dengue transmission; systematic review. Tropical Med Int Health.

[41] Bowman LR, Donegan S, McCall PJ (2016). Is dengue vector control deficient in effectiveness or evidence? Systematic review and meta-analysis. PLoS Negl Trop Dis.

[42] Barrera R, Acevedo V, Felix GE, Hemme RR, Vazquez J, Munoz JL, Amador M (2017). Impact of autocidal gravid ovitraps on Chikungunya virus incidence in *Aedes aegypti* (Diptera: Culicidae) in areas with and without traps. J Med Entomol.

[43] Eiras AE, Buhariar TS, Ritchie SA (2014). Development of the gravid *Aedes* trap for the capture of adult female container-exploiting mosquitoes (Diptera: Culicidae). J Med Entomol.

