# Supplementary figures and images for: Effectiveness of a field trap barrier system for controlling Aedes albopictus: a “removal trapping” strategy
Source: Parasit Vectors. 2018 Feb 20;11:101. doi: 10.1186/s13071-018-2691-1 (PMC5819175; doi:10.1186/s13071-018-2691-1)

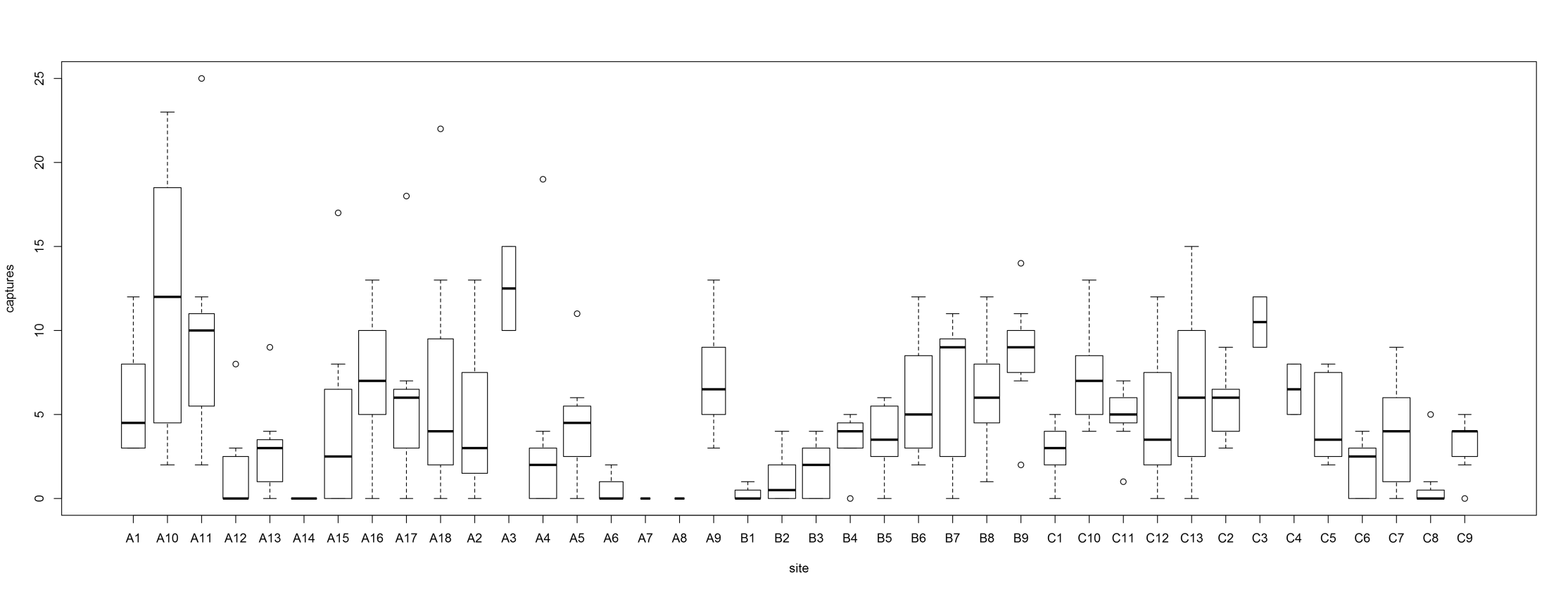

Supplement: Supplementary file 1 — Figure S1. Variations in daily captures of Ae. albopictus among traps. (TIFF 102 kb) [file 13071_2018_2691_MOESM1_ESM.tif]

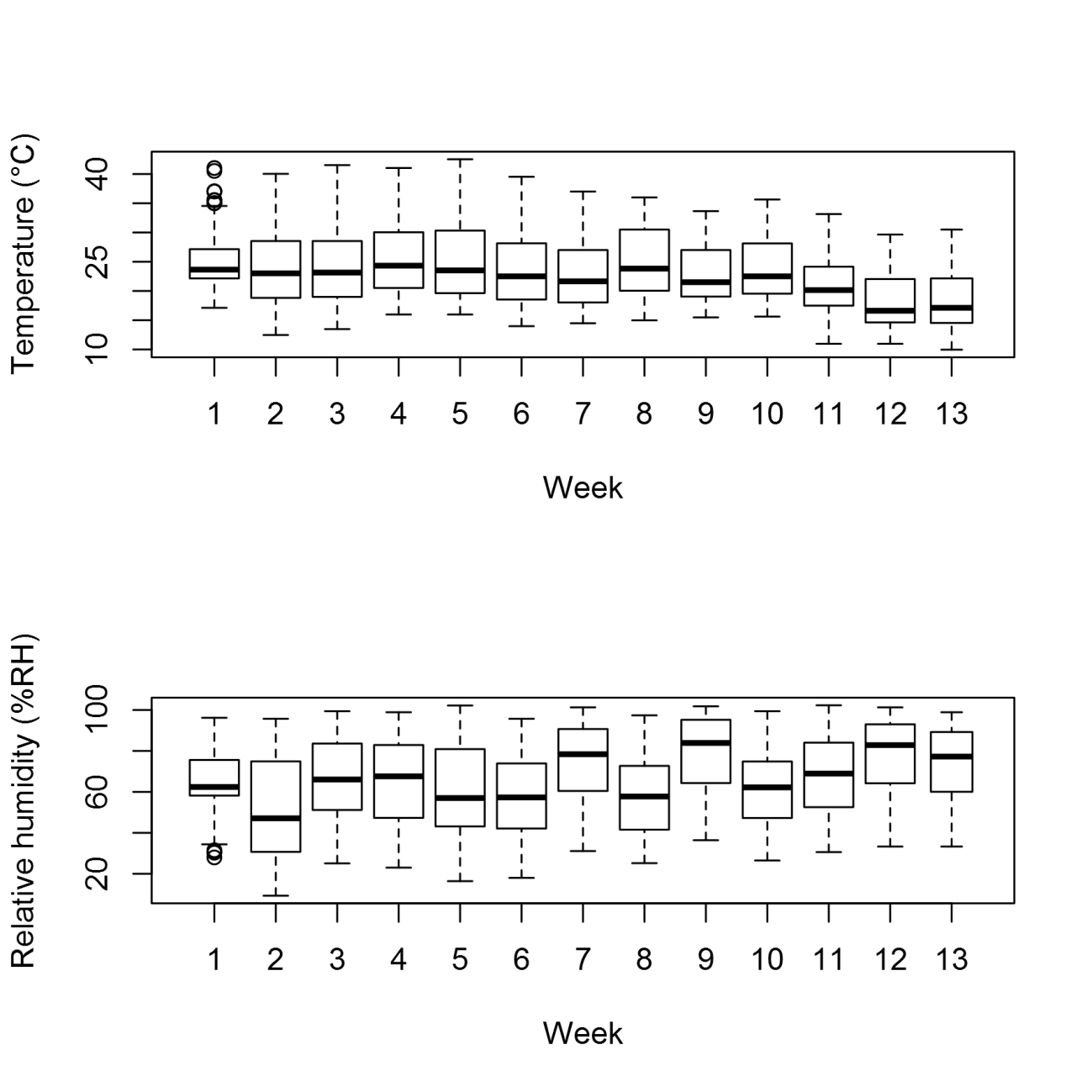

Supplement: Supplementary file 2 — Figure S2. Variations in daily temperature and relative humidity recorded weekly by data loggers. (TIFF 111 kb) [file 13071_2018_2691_MOESM2_ESM.tif]
